# Corticosteroids for treatment of COVID-19: effect, evidence, expectation and extent

**DOI:** 10.1186/s43088-021-00165-0

**Published:** 2021-11-04

**Authors:** Vijay K. Patel, Ekta Shirbhate, Preeti Patel, Ravichandran Veerasamy, Prabodh C. Sharma, Harish Rajak

**Affiliations:** 1 Institute of Pharmaceutical Sciences, Guru Ghasidas University, Bilaspur, C.G. 495 009 India; 2 Faculty of Pharmacy, AIMST University, 08100 Semeling, Bedong, Kedah Darul Aman Malaysia; 3 Department of Pharmaceutical Chemistry, Delhi Pharmaceutical Sciences and Research University, MB Road, Pushp Vihar, New Delhi, 110 017 India

**Keywords:** Corticosteroids, COVID-19, Dexamethasone, Methylprednisolone, Hydrocortisone, Prednisone

## Abstract

**Background:**

The World Health Organization (WHO) announced the COVID-19 occurrence as a global pandemic in March 2020. The treatment of SARS-CoV-2 patients is based on the experience gained from SARS-CoV and MERS-CoV infection during 2003. There is no clinically accepted therapeutic drug(s) accessible yet for the treatment of COVID-19.

**Main body:**

Corticosteroids, i.e., dexamethasone, methylprednisolone, hydrocortisone and prednisone are used alone or in combination for the treatment of moderate, severe and critically infected COVID-19 patients who are hospitalized and require supplemental oxygen as per current management strategies and guidelines for COVID-19 published by the National Institutes of Health. Corticosteroids are recorded in the WHO model list of essential medicines and are easily accessible worldwide at a cheaper cost in multiple formulations and various dosage forms. Corticosteroid can be used in all age group of patients, i.e., children, adult, elderly and during pregnancy or breastfeeding women. Corticosteroids have potent anti-inflammatory and immunosuppressive effects in both primary and secondary immune cells, thereby reducing the generation of proinflammatory cytokines and chemokines and lowering the activation of T cells, monocytes and macrophages. The corticosteroids should not be used in the treatment of non-severe COVID-19 patients because corticosteroids suppress the immune response and reduce the symptoms and associated side effects such as slow recovery, bacterial infections, hypokalemia, mucormycosis and finally increase the chances of death.

**Conclusion:**

Intensive research on corticosteroid therapy in COVID-19 treatment is urgently needed to elucidate their mechanisms and importance in contributing toward successful prevention and treatment approaches. Hence, this review emphasizes on recent advancement on corticosteroid therapy for defining their importance in overcoming SARS-CoV-2 pandemic, their mechanism, efficacy and extent of corticosteroids in the treatment of COVID-19 patients.

## Background

The World Health Organization (WHO) announced the coronavirus disease 2019 (COVID-19) occurrence as a global pandemic in March 2020. COVID-19 is an infectious disease caused by severe acute respiratory syndrome coronavirus 2 (SARS-CoV-2) that appeared from Wuhan, Hubei province, China in Dec 2019 [1, 2]. The most frequent indications of COVID-19 infection include fever, cough and myalgia. The infected persons have some additional symptoms like diarrhea, nasal congestion, respiratory distress, sepsis, septic shock, loss of smell and taste [3–5].

The crown-like spikes on the outer surface representing corona were responsible for naming the virus as ‘Coronavirus.’ The causative virus belongs to the family, Coronaviridae, and order, Nidovirales; bearing a single-stranded genomic RNA [6]. The SARS-CoV, SARS-CoV-2, H5N1 influenza, H1N12009 and Middle East Respiratory Syndrome Coronavirus (MERS-CoV) were earlier thought to infect only animals, but with the outbreak of 2003 SARS-CoV in China and MERS-CoV in Middle Eastern countries, the world witnessed its infectious ability in humans, causing severe pulmonary failure and sometimes fatality. The possible evidence of animal to human transmission of coronavirus was also established in the COVID-19 pandemic [7–9]. However, the scientific studies have reported human to human spread of SARS-CoV-2 via droplets or direct contact. The possibility of transmission increases rapidly through asymptomatic carriers [10].

The current treatment of SARS-CoV-2 infected persons is based on the experiences gained from SARS-CoV and MERS-CoV infection during 2003 along with the application of existing antiviral/antibiotic therapy and mechanical respiratory support [11]. Remdesivir is the only FDA-authorized investigational drug in the treatment of COVID-19 that has displayed activity against SARS-CoV-2, significantly reducing the time to recovery and mortality, while not shown clinical improvement after 5 days and have no statistically significant effect on deaths. It is presently accessible in an inadequate number of pharmacies and hospitals around the world [12]. There are no FDA-approved therapeutic medicines available yet for the treatment of COVID-19 [13]. There is an urgent need for an effective drug for the treatment of COVID-19 patients which should be available globally at a low cost, reducing the recovery time and death rate of infected patients.

## Main text

### Corticosteroids in COVID-19 treatment

**Fig. 1:**
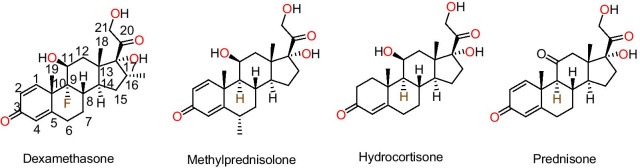
Chemical structure of corticosteroids being evaluated as potential treatment of COVID-19 patients

Corticosteroids are a class of natural, semi-synthetic and synthetic steroidal hormones that are produced in the adrenal cortex of vertebrates and are required for a variety of physiological functions such as immune response, stress response, control of inflammation, protein catabolism, carbohydrate metabolism and blood electrolyte concentrations [14]. Corticosteroids, alone or in combination with ribavirin, were particularly efficient in the treatment of the SARS outbreak in 2003 [15, 16]. Various clinical studies have reported that corticosteroids, i.e., dexamethasone, prednisone, methylprednisolone and hydrocortisone (Fig. 1) can be used for the treatment of mild, moderate, severe and critically ill COVID-19 patients (Table 1). Most of the time, the death of COVID-19 patients is occurring due to an overactive immune response to the infection not due to the coronavirus. The corticosteroids control the immune system or cytokine storm, providing support for patients, whose lungs are damaged due to an overactive immune response that an occasionally found in the severe incidents of COVID-19. Nevertheless, corticosteroids have exhibited detrimental effects, including extended time for viral clearance and a higher possibility of infection. Corticosteroids decrease the time required on mechanical ventilation, the residing period in an intensive care unit (ICU) and importantly death rate in COVID-19 patients [17]. Corticosteroids can be used in all age groups of patients, i.e., children, adults, the elderly and during pregnancy or breastfeeding women. The recovery trial employed oral and intravenous (IV) infusion of dexamethasone to COVID-19 patients, including pregnant or breastfeeding women [18]. Corticosteroids are the part of WHO model list of essential medicines and are easily accessible worldwide at a cheaper cost, in multiple formulations and various dosage forms, such as tablet, injectable suspension, oral solution, oral concentrate and administered orally, intramuscular, intra-articular, direct IV injection or intra-lesional injections and inhalation. The corticosteroids can be administered by inhalation in patients suffering from asthma and chronic obstructive pulmonary disease (COPD) [19]. Inhaled corticosteroids studies are going on in various clinical trials and still need to be studied for assessing their efficacy in COVID-19 [20]. Intensive research on corticosteroid therapy in COVID-19 treatment is urgently needed to elucidate their mechanisms and importance in contributing toward successful prevention and treatment approaches. Hence, this review emphasizes on recent advancement on corticosteroid therapy for defining their importance in overcoming SARS-CoV-2 pandemic, their mechanism, efficacy and extent of corticosteroids in the treatment of COVID-19 patients.

**Table 1:** An overview of clinical trials: corticosteroids as potential treatment of COVID-19

Clinical trail/drug	Condition or disease	Dose	Outcome	References
RECOVERY (NCT04381936)/Low dose, Dexamethasone	Suspected or confirmed COVID-19	6 mg PO/IV daily × 10 days	Remarkable result on critically ill patients. No effect on mortality in less severe cases of COVID-19	[23, 24]
CoDEX (NCT04327401)/ High Dose, Dexamethasone	COVID-19 moderate or severe ARDS	20 mg IV daily × 5 days, then 10 mg IV daily × 5 days or until ICU	Increased efficacy and not showed significant adverse effect from the treatment	[25, 26]
DEXA-COVID19 (NCT04325061)/High Dose, Dexamethasone	Mechanically ventilated, moderate-severe ARDS, confirmed COVID-19	20 mg IV daily × 5 days, then 10 mg IV daily × 5 days	60-day mortality with side effect	[27]
Low dose Methylprednisolone	Severe patients with COVID-19 pneumonia	2 mg/kg IV daily × 5–7 days	Length of ICU hospitalization was significantly shorter while not showed significant difference of mortality rate	[32]
High dose Methylprednisolone	Critical patients with COVID-19	Single dosage 40–500 mg according to severity	Improved lung function without negative impacts on the production of specific IgG antibody against coronavirus SARS-CoV-2	[33]
High dose Methylprednisolone	COVID-19 pneumonia	1 mg/kg IV daily × 7 days	No significant results were observed in 14 days. After 14 days improved or alleviated clinical symptoms and signs	[34]
High dose Methylprednisolone	Critical patients with COVID-19 pneumonia	40 mg IV two times daily × 3 days, then 20 mg IV two times daily × 3 days	Beneficial effect and decreasing the risk of admission to ICU, NIV or death	[35]
MetCOVID (NCT04343729)/High dose Methylprednisolone	Hospitalized patients with clinical and/or suspected COVID-19	0.5 mg/kg IV two times daily × 5 days	No reduction in mortality. Sepsis or positive blood culture collected on day 7	[36, 37]
Steroids-SARI (NCT04244591/High dose Methylprednisolone	COVID-19 patients with severe acute respiratory failure	40 mg IV Single dosage × 5 days	Lower lung injury at 7–14 days, Secondary bacterial infections; barotrauma; severe hyperglycemia; GI bleeding; attained disability	[38]
COVID STEROID (NCT04348305)/ Low dose, Hydrocortisone	COVID-19 and severe hypoxia	200 mg IV daily × 7 days	Initially patients not require life support; After 28 days, serious adverse effects observed	[17, 41]
REMAP-CAP (NCT02735707)/Low dose, Hydrocortisone	Critically ill COVID-19 patients with acute respiratory failure	200 mg IV daily × 7 days, then 100 mg IV daily × 4 days, then 50 mg IV daily × 3 days	Did not considerably decrease the infection, at day 21 treatment failure observed	[42]
CAPE-COVID (NCT02517489)/Low dose, Hydrocortisone	Minimal severity: admitted to ICU or intermediate care unit, on oxygen probable or confirmed COVID-19	Hydrocortisone IV continuous infusion × 8 or 14 days (200 mg IV daily × 4 or 7 days, 100 mg IV daily × 2 or 4 days, 50 mg IV daily × 2 or 3 days)	No significant difference in rate of treatment failure between hydrocortisone and placebo group observed in 21 days	[43]

### Dexamethasone

Dexamethasone (MK-125) is a synthetic corticosteroid developed in 1957. Its chemical structure contains the cyclopentaphenanthrene ring substituted by fluorine at position 9, substituted by hydroxy groups at positions 11, 17 and 21, methyl group at position 10, 13 and 16 and oxo groups at positions 3 and 20 (Fig. 1). Dexamethasone is used in the treatment of many conditions, including different inflammatory conditions, immune system disorders such as immunosuppressive, allergic disorders, asthma, chronic obstructive lung disease, rheumatic problems, inflammation in brain, croup, eye pain following eye surgery, tuberculosis bowel disorders and some cancers. Dexamethasone has been a part of the WHO model list of essential medicines since 1977 in the form of numerous formulations with various dosage forms such as tablet, injectable suspension, oral solution and oral concentrate, readily available globally at a low cost and administered orally, intramuscular, intra-articular or direct IV injection [21, 22].

Horby et al. performed controlled trials of corticosteroids as a potential treatment of COVID-19 in March 2020 called RECOVERY trial, for testing a range of potential therapies (Table 1). The dexamethasone 6 mg/day was either administered orally or IV injection for 10 days for randomly selected 2104 patients and compared with 4321 patients who concurrently received standard care for COVID-19 infection [23, 24]. During these studies, dexamethasone showed the remarkable results in critically ill patients who received mechanical ventilation by ventilators and who received oxygen therapy. Dexamethasone declined mortality by 33% in patients taking invasive mechanical ventilation and 20% in patients getting oxygen lacking invasive mechanical ventilation. The application of dexamethasone showed no effect in less severe cases of COVID-19 and has not reduced mortality in these patients [23, 24]. The treatments were comprised of short duration, recommended doses by doctors, and no serious side effects were observed, the potentially higher blood glucose level (hyperglycemia) was a temporary condition. However, prolonged application, i.e., more than 2 weeks has been found to produce undesirable events namely, glaucoma, hypertension, fluid retention, cataract, weight gain, elevated threat of infections, osteoporosis and psychological effects, e.g., mood swings, confusion or irritation and memory issues [18, 23, 24].

Tomazini et al. studied the effect of dexamethasone on mild ill COVID-19 patients who do not receive mechanical ventilation nor associated with Moderate or Severe Acute Respiratory Distress Syndrome (ARDS). The continuous IV infusion of dexamethasone 20 mg/daily was administered for 5 days subsequently 10 mg/daily for 5 days or until ICU discharge, in addition to standard care. This treatment enhanced the efficacy and significantly no adverse effect was observed (Table 1) [25, 26].

Dr. Negrin University Hospital studied the effectiveness of dexamethasone in patients with ARDS caused by COVID-19 under a clinical trial named DEXA-COVID19 with 200 participants in Spain. An IV dose of dexamethasone, i.e., 20 mg/day for the first 5 days, subsequently 10 mg/day for the next 6–10 days was administered. The primary outcome was 60-day mortality and the mortality outcome was 28 days, when compared with standard care as control of intervention (Table 1) [27].

### Methylprednisolone

Methylprednisolone is a synthetic corticosteroid first described in the literature in 1950 [28, 29]. The chemical structure contains the cyclopentaphenanthrene ring substituted by hydroxy groups at positions 11, 17 and 21, methyl group at positions 6, 10, 13 and oxo groups at positions 3 and 20 (Fig. 1). Methylprednisolone is being used for the treatment of many conditions like endocrine, ophthalmic, respiratory, rheumatic, skin disorders, blood disorders, fluid retention in body tissues, alopecia areata and discoid lupus erythematosus. Methylprednisolone has been recorded in the WHO model list of Essential Medicines in numerous formulations with various dosage forms such as tablet, injectable suspension, oral solution, oral concentrate, readily available globally at a low cost and administered orally, intramuscular, intra-articular, direct IV injection or intra-lesional injections. Methylprednisolone is the second drug in the class of corticosteroids, which has shown promising results in lowering mortalities after successful use of dexamethasone in critically ill COVID-19 patients [22, 30].

Wang et al. studied the application of methylprednisolone as possible treatment in severe patients with COVID-19 pneumonia. The IV injection of methylprednisolone 2 mg/kg/day for 5–7 days was administered to the patients. These studies showed that the application of methylprednisolone reduces the length of ICU hospitalization, while it did not show significant difference in death rate of COVID-19 patients (Table 1) [31].

Liu et al. studied the effect of methylprednisolone in severe and critically ill COVID-19 patients. A single dosage of 40–500 mg methylprednisolone as per the harshness of COVID-19 improved the lung performance without decreasing the generation of specific IgG antibodies against SARS-CoV-2 infection, while successfully halting the inflammatory cascade (Table 1) [32].

Beijing Chao Yang Hospital studied the efficacy and safety of methylprednisolone therapy against new coronavirus pneumonia. The dosage schedule was IV injection of methylprednisolone 1 mg/kg/day for 7 days. This study employed a lower dose of methylprednisolone, which did not show significant improvement in patient conditions during first 14 days. After 14th day, recovery in patient condition along with alleviation of clinical symptoms and signs was noticed and the patient did not require any additional or alternative treatment (Table 1) [33].

Corral et al. evaluated the efficacy of methylprednisolone in serious patients with COVID-19 pneumonia. The dosage schedule employed was IV infusion of methylprednisolone 40 mg/12 h for 3 days, after that 20 mg/12 h for 3 days. This 6 days course of methylprednisolone showed a beneficial effect along with decreased risk of admission to ICU, NIV or death (Table 1) [34].

Jeronimo et al. evaluated the efficacy of methylprednisolone in suspected COVID-19 infected persons with IV infusion of methylprednisolone in dose of 0.5 mg/kg twice in a day, for 5 days. This short course treatment schedule of methylprednisolone was not able to reduce mortality in COVID-19 patients (Table 1) [35, 36].

Peking Union Medical College hospital carried out clinical trial comprising of 80 patients for evaluating the effectiveness and safety of methylprednisolone in COVID-19 critically ill patients with Severe Acute Respiratory Failure. The dosage schedule was comprised of IV administration of 40 mg methylprednisolone every 12 h for 5 days. The outcome of studies was lesser lung injury at 7 and 14 days as determined using Murray lung injury score. The significant adverse effects noticed were secondary bacterial infections, severe hyperglycemia, GI bleeding and attained disability (Table 1) [37].

### Hydrocortisone

Hydrocortisone (cortisol or 17-hydroxycorticosterone) is a synthetic or semi-synthetic analog of naturally occurring glucocorticoid produced by the adrenal cortex, discovered in the 1930s by Edward Kendall. The chemical structure contains the cyclopentaphenanthrene ring, substituted by hydroxy groups at positions 11, 17 and 21, methyl group at positions 10, 13 and oxo groups at position 3 (Fig. 1). Hydrocortisone is employed in the treatment of inflammation, allergy, asthma, adrenocortical deficiency and few cancerous circumstances [22, 30].

Petersen et al. performed the clinical trial to investigate the effects of low-dose hydrocortisone in COVID-19 and critical hypoxic patients not supplied with life-support system, i.e., mechanical ventilation, circulatory support or renal replacement therapy. The continuous IV infusion of hydrocortisone 200 mg/daily was administered for 7 days beside the standard care. After 28 days, critical side effects, i.e., anaphylactic reaction, invasive fungal infection and gastrointestinal hemorrhage were observed (Table 1) [17, 38].

REMAP-CAP investigators reported the consequences of hydrocortisone on death rate and organ support in patients with severe infection of COVID-19 using data from 121 sites spread over 8 countries. The hydrocortisone was administered IV in the dose of 50 mg/day for 7 days or 100 mg every 6 h for 7 days, a shock-dependent course comprising of 50 mg every 6 h for 7 days when the shock was clinically apparent. This treatment employing hydrocortisone, when compared with no hydrocortisone, showed 93% and 80% chances of progress in organ support free days within 21 days (Table 1) [39–41].

Dequin et al. reported multicenter randomized double-blind clinical trial performed in France to examine the effect of hydrocortisone in seriously ill COVID-19 persons for monitoring the 21-day death rate or respiratory support requirement. These studies were carried out to find out hydrocortisone as a possible therapeutic option for treatment of severe lung damage occurring in COVID-19. The IV infusion of hydrocortisone 200 mg/daily was administered for 7 days followed by decreased dose to 100 mg/day for 4 days and 50 mg/day for 3 days, for a total of 14 days. This clinical trial employing low dose hydrocortisone, when compared with placebo did not significantly reduce the infection on day 21 (Table 1) [42, 43].

### Prednisone

Prednisone is a synthetic glucocorticoid analog of cortisone. The chemical structure contains the cyclopentaphenanthrene ring, substituted by hydroxy groups at positions 17 and 21, a methyl group at positions 10, 13 and oxo groups at positions 3, 11 (Fig. 1). Prednisone is used for its immunosuppressive activity in allergic, skin, blood, gastrointestinal, nervous system, kidney, respiratory disorders and organ transplantation [22, 30].

Keller et al. [44] reported that glucocorticoid formulations of prednisone can give the same life-saving benefits in COVID-10 treatment as dexamethasone, methylprednisolone and hydrocortisone (Table 1). Bozzette et al. [45] reported that prednisone used in patients with pneumonia and hypoxia reduces the risk of death (Table 1).

### The combination of corticosteroids with other drugs for treatment of COVID-19 infection

#### Corticosteroids plus Remdesivir

The dexamethasone plus remdesivir combination is used to treat hospitalized patients, who require increasing amounts of supplemental oxygen, high-flow oxygen or noninvasive ventilation, but it is not recommended in patients who require invasive mechanical ventilation or extracorporeal membrane oxygenation (ECMO) [46]. The safety and efficacy of the combination of dexamethasone and remdesivir have not been adequately examined in clinical studies; consequently, there is insufficient clinical data to justify the use of this combination medication. The combination of dexamethasone and remdesivir is recommended because there are theoretical considerations, according to scientists, that corticosteroids have potent anti-inflammatory effects to prevent hyperinflammatory effects and that remdesivir has an antiviral effect. The combination may treat the viral infection and dampen the potentially injurious inflammatory response that is a consequence of the infection. However, the data on clinical outcomes for patients who received this combination are currently limited [50]. If dexamethasone isn't accessible, other corticosteroids like prednisone, methylprednisolone or hydrocortisone can be used in place of it.

#### Corticosteroids plus Tocilizumab

The dexamethasone or another corticosteroid at an equivalent dose plus tocilizumab combination is used for the treatment of recently hospitalized patients who require supplemental oxygen, invasive mechanical ventilation or ECMO have either been admitted to the ICU within the prior 24 h or have significantly increased inflammatory markers of inflammation [46]. The COVID-19 viral infection of some severe patients suffers from cytokine storm syndrome (CSS) causing hypotension, fever and high levels of C-reactive protein (CRP), abnormal coagulation, uncontrolled inflammatory response, shock and multiorgan system failure. The patients suffer CSS elevated levels of cytokines thus raising interleukin-6 (IL-6), interleukin-10 (IL-10) and interferon-gamma (IFN-γ). The scientific reports indicate that the SARS-CoV-2 infection is associated with a significant increase in IL-6 [51, 52]. Tocilizumab is a monoclonal antibody that inhibits IL-6, and the FDA has already granted emergency use authorization for the management of COVID-19 related CSS and organ failure. Sarilumab is another IL-6 inhibitor that has been recommended as a replacement for tocilizumab [46].

#### Corticosteroid plus Baricitinib

The combination of dexamethasone or another corticosteroid at an equivalent dose plus baricitinib is used for recently hospitalized patients (within 3 days of hospital admission) who have rapidly increasing oxygen needs, require high-flow oxygen or noninvasive ventilation and have increased markers of inflammation, as well as selected patients, who are on low-flow oxygen but are progressing toward needing higher levels of respiratory support [46]. Baricitinib is an oral Janus kinase (JAK) inhibitor for the treatment of rheumatoid arthritis and granted an emergency use authorization for the treatment of COVID-19 in the USA. Baricitinib has immunomodulatory, antiviral effects and is able to prevent the poorly regulated production of proinflammatory cytokines in severe or critical COVID-19 patients [53]. The baricitinib in combination with tocilizumab for the treatment of COVID-19 is strictly prohibited because both are potent immunosuppressants, there is the potential for an additive risk of infection. If baricitinib isn't accessible, tofacitinib, another JAK inhibitor, could be used instead [46, 53].

### Current management strategies and guidelines for the use of corticosteroids in COVID-19

The National Institutes of Health (NIH) has published treatment guidelines for COVID-19 that includes the use of corticosteroids. The patients with asymptomatic/presymptomatic and mild infection of COVID-19 exhibit a variety of symptoms such as fever, sore throat, cough, loss of taste and smell, headache, muscle pain, malaise, nausea, vomiting and diarrhea; The corticosteroids should not be used in asymptomatic/ presymptomatic and mild infection of COVID-19 (Table 2). The COVID-19 patients already receiving dexamethasone or another corticosteroid may suffer from another disease including different inflammatory conditions, immune system disorders as immunosuppressive, allergic disorders, asthma, chronic obstructive lung disease, rheumatoid arthritis (RA), inflammation in brain, croup, eye pain following eye surgery, tuberculosis bowel disorders and some cancers **s**hould continue therapy as directed by their treatment center [46].

**Table 2:** Guidelines for the use of corticosteroids in COVID-19 patients

S. No.	Class of COVID-19	Symptom	Corticosteroids treatment guideline/protocol
01	Asymptomatic/Presymptomatic Infection of COVID-19	No symptoms	Do not use corticosteroids The patients suffer from other disease already receiving dexamethasone or another corticosteroid **s**hould continue therapy as directed by doctor
02	Mild Infection of COVID-19	Fever, cough, sore throat, malaise, headache, muscle pain, nausea, vomiting, diarrhea, loss of taste and smell	Do not use corticosteroids The patients suffer from other disease already receiving dexamethasone or another corticosteroid **s**hould continue therapy as directed by doctor
03	Moderate Infection of COVID-19	Lower respiratory disease (SpO_2_). 90% to < 93% on room air at sea level. Respiratory rate > 24/min, breathlessness but not require supplemental oxygen High-risk patients at deterioration requiring hospitalization and supplemental oxygen	Isolation is necessary. Do not use corticosteroids Dexamethasone 6 mg/Prednisone 40 mg/Methylprednisolone 32 mg/Hydrocortisone 160 mg Dexamethasone plus remdesivir
04	Severe Infection of COVID-19	Obstructed or absent breathing, severe respiratory distress, shock, coma and/or convulsions. The patients have SpO_2_ < 90% on room air at sea level. The patients treated with high-flow nasal oxygen (HFNO) systems or noninvasive ventilation The patients who have recently hospitalized rapidly increasing oxygen needs, require high-flow oxygen or noninvasive ventilation and have increased markers of inflammation	Dexamethasone 6 mg/Prednisone 40 mg/Methylprednisolone 32 mg/Hydrocortisone 160 mg Dexamethasone plus remdesivir Add baricitinib or tocilizumab (within 3 days of hospital admission) one of the two options above
05	Critical Infection of COVID-19	Respiratory failure, septic shock and/or multiple organ dysfunction. Patients who require invasive mechanical ventilation or extracorporeal membrane oxygenation	Dexamethasone 6 mg/Prednisone 40 mg/Methylprednisolone 32 mg/Hydrocortisone 160 mg Dexamethasone plus Tocilizumab for patients who are within 24 h of admission to the ICU

The patients with moderate infection of COVID-19 have the lower respiratory disease during clinical assessment or imaging and an oxygen saturation (SpO_2_) 90% to < 93% on room air at sea level, respiratory rate > 24/min, breathlessness but not require supplemental oxygen. It is advised not to use corticosteroids for the treatment of COVID-19 patients [46]. However, the patients who require supplemental oxygen but who do not require oxygen delivery through a high-flow device, noninvasive ventilation can be treated with dexamethasone 6 mg once daily plus standard of care for up to 10 days or until hospital discharge, whichever came first. If dexamethasone is not available, an alternative corticosteroid such as prednisone 40 mg/methylprednisolone 32 mg administered once daily or in two divided doses daily, or hydrocortisone 160 mg administered in two to four divided doses daily can be used (Table 2) [54]. The combination of dexamethasone and remdesivir for anti-inflammatory effects and antiviral effect, respectively, is recommended as a treatment option for patients of this group [46].

The patients with COVID-19 are considered to have severe infection of COVID-19, if they have SpO2 < 90% on room air at sea level, a respiratory rate > 30 breaths/min, PaO2/FiO2 < 300 mm Hg or lung infiltrates > 50%. These patients may experience rapid clinical deterioration. Oxygen should be administered immediately through a high-flow device or noninvasive ventilation but not invasive mechanical ventilation or extracorporeal membrane oxygenation. The severe infection of COVID-19 should be treated with dexamethasone alone or an alternative corticosteroid such as prednisone/ methylprednisolone/ hydrocortisone or dexamethasone plus remdesivir. The COVID-19 patients, who have been recently hospitalized with rapidly increasing oxygen needs, require high-flow oxygen or noninvasive ventilation and have increased markers of inflammation, within 3 days of hospital admission should be treated with baricitinib or tocilizumab plus dexamethasone alone or baricitinib or tocilizumab plus dexamethasone plus remdesivir (Table 2). The combination of baricitinib with tocilizumab for the treatment of COVID-19 is strictly prohibited as both the drugs are potent immunosuppressants and may have a potential for an additive risk of infection [46].

The critical infection of COVID-19 patients may have acute respiratory distress syndrome, septic shock, cardiac dysfunction, an exaggerated inflammatory response and/or multiple organ dysfunction. The patients who require invasive mechanical ventilation or extracorporeal membrane oxygenation are treated with dexamethasone alone. If dexamethasone is not available, equivalent doses of alternative corticosteroids prednisone, methylprednisolone or hydrocortisone may be used. The patients who are admitted within 24 h of admission to the ICU are treated with dexamethasone plus tocilizumab (Table 2) [46].

Inhaled corticosteroids (ICS) are a major course of treatment for asthma patients. Corticosteroids may have two major effects, (a) they can prevent excessive inflammation in the host, including cytokine storming, and (b) they can also induce coronavirus multiplication by suppressing innate immunity. As per WHO guidelines, systemic corticosteroids should be administered with caution and only in certain situations. Several recent Japanese researches have shown that ciclesonide, a form of ICS, may be beneficial in the treatment and prognosis of COVID-19 patients with asthma [47, 48]. The worldwide asthma guideline, Global Initiative for Asthma (GINA), advises that asthma sufferers should keep on using ICS and oral corticosteroids, even during the COVID-19 pandemic period. Yet, no research supporting this recommendation has been published so far. One of the independent studies revealed that administration of asthma medicine had no prognosis effect on COVID-19 clinical outcomes and that further research is required [49].

### Mode of action of corticosteroids in COVID-19 treatment

Though mainstream patients endure an uneventful reclamation, around 19% patients show progressive worsening with severe pneumonia [2], followed by severe dyspnea, ARDS, multiple organ dysfunction and sometimes death [55, 56]. The condition worsens with the unstoppable arrival of ‘cytokine storm’ in infected individuals. Earlier studies on SARS-CoV and MERS-CoV infected patients show an excessive generation and release of proinflammatory cytokines and chemokines like tumor necrosis factor-α, IL-1, IL-2, IL-6, IL-8, IL-12, INF-α, INF-β, INF-γ, monocyte chemoattractant protein 1 (MCP1), INF-γ-induced protein 10 (IP10) [12] in their serological findings. The similar findings were also evident in individuals with COVID-19.

Initially, with the invasion of SARS-CoV-2 through the angiotensin-converting enzyme 2 receptor in the body, the virus vigorously replicates infecting airway and alveolar epithelial cells at the prior stage. This stimulates the specific immune cells (like monocytes, T cells, B cells, natural killer cells) promoting colossal production of cytokines and chemokines, hence bringing ‘cytokine storm.’ The cytokine storm upholds the bulk movement of inflammatory cells into the lungs, accompanying with enormous vascular inflammation, shock and hypotension, causing multi-organ failure and death. On one end, the balanced immune response of the body tries to keep and manage the infection under control, but on contrary, the exaggerated immune response favors pulmonary damage and failure [12, 57]. The involvement of immune response in COVID-19 pathophysiology raised a thought in the researcher’s mind that intervention of corticosteroids in COVID-19 therapy may prove to be a beneficial and life-saving measure for critically infected individuals from this global pandemic.

Corticosteroid treatment may serve as a sailor for drowning life of patients with severe pneumonia in presence of COVID-19. Though it’s an immune-suppressive property impairing the innate immunity may worsen the intensity of viral propagation. Conversely, patients undergoing long term maintenance dose of steroids exhibits attained encouraging outcome without any increased occurrence of development of serious COVID-19 pneumonia [58]. Therefore, caution should be adopted before including corticosteroids in the COVID-19 treatment regimen.

**Fig. 2:**
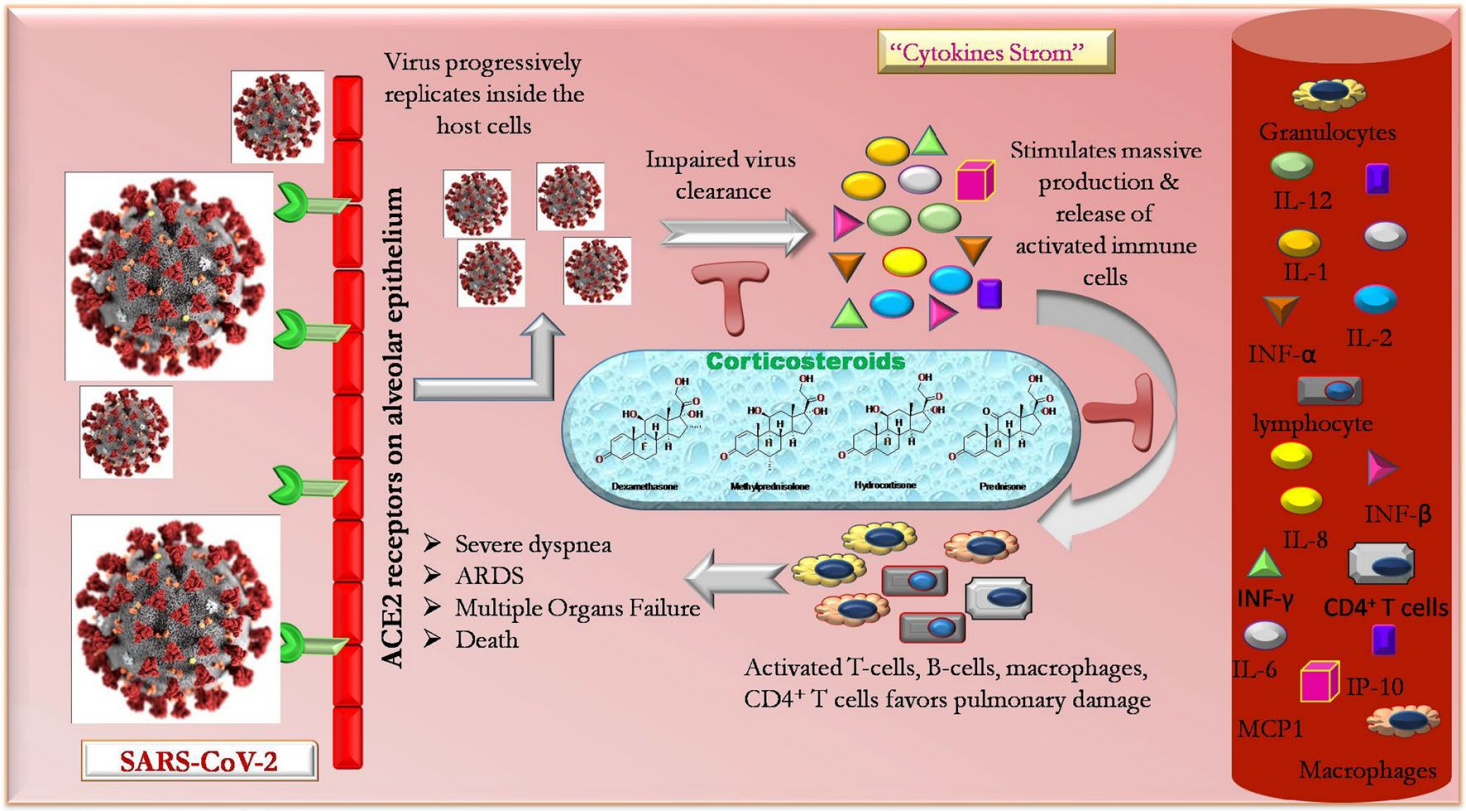
Mechanism of action of corticosteroids in treatment of COVID-19

The count of specific immune cells like macrophages, monocytes, natural killer cells, T cells, B cells, etc. is markedly affected in individuals with COVID-19. Lymphopenia seems to be a precarious factor related to disease harshness and modality. The results of a pilot study conducted by Guo and co-workers revealed a noticeable decrease in the number of CD3^+^ T cells, CD3^+^ CD4^+^ T cells and CD3^+^ CD8^+^ T cells in the patients who died with viral pneumonia. The results suggested that the cellular immune function of such individuals was significantly inhibited. The CD4^+^ T cells are responsible for maintaining immunity through T cells antiviral response. The researches even showed that an increased concentration of both extremely proinflammatory CCR6^+^ Th17 in CD4 T cells and cytotoxic granules in CD8 T cells. The over-activated T cells severely cause immune injury accompanied with elevated Th17 and high cytotoxicity of CD8 T cells. In such individuals, corticosteroids usage may hinder T cells immunity and result in tenacious viral replication and subsequent delay in clearance [57]. Systemic corticosteroids appear to aggravate the viral load due to their immune-suppressive effect and delay the viral excretion. Nevertheless, recent reports highlight the fact that corticosteroids may possess a stimulatory as well as inhibitory action on the immune response depending upon the duration and their blood concentration [59]. The possible mechanism of action of corticosteroids in COVID-19 treatment is depicted in Fig. 2.

### Caution against corticosteroid-based COVID-19 treatment

The corticosteroids should be used under the supervision of a clinician for the treatment of COVID-19 patients. The use of corticosteroid in the treatment of COVID-19 treatment is controversial, some scientific reports do not support corticosteroid treatment due to the huge side effects such as increased mortality [60, 61], obstructed clearance of viral RNA from the respiratory tract [57], slow clearance of viral RNA from blood [62], psychosis [63], induced diabetes [64], avascular necrosis in survivors [65], induced lung injury or shock [66]. However, some scientific reports support the application of corticosteroids at low-to-average amount for a shorter period as it declines the death rate and minimizes the duration of hospitalization for seriously ill patients with COVID-19 without causing secondary infection and other side effects (Table 1) [17, 67]. The RECOVERY trial noticed a noteworthy decline in death with dexamethasone only in serious cases of COVID-19 [23, 24]. The WHO published guidance on September 02, 2020, regarding the use of corticosteroids in patients with COVID-19. According to these guidelines, corticosteroids are recommended for the treatment of patients with severe and critical COVID-19. The corticosteroids should not be used in the treatment of non-severe COVID-19 patients because corticosteroids suppress the immune response and reduce the symptoms and associated side effects such as slow recovery, bacterial infections, hypokalemia and finally increase the chances of death [61]. Salinas et al. and Saiz-Rodríguez et al. studied the effect of early use of corticosteroids in non-critical COVID-19 patients, the primary result indicated serious side effect to patients such as respiratory deterioration, worsening of the glycemic profile, delirium, pneumonia, urinary tract infection, device-associated infections, need to admission to ICU, mechanical ventilation or death by day 28. The COVID-19 pandemic has been reported with very high incidence of mucormycosis (black fungus infection) among COVID-19 patients, especially in those who have had treatment with higher doses or longer courses of steroids or who are diabetic or cancer or HIV or have weak immune systems and have received steroids [68, 69].

The corticosteroids can intensify the possibility of osteonecrosis of the femoral head (ONFH) and it should be prevented by avoiding their use and/or minimizing the dose and duration of corticosteroids. The COVID-19 patients being treated with corticosteroids should also be prescribed with biphosphates and vitamin E. The latter drugs could also be replaced with vasodilators, anticoagulants or traditional Chinese medicine [70, 71].

## Conclusions

The COVID-19 pandemic is uncontrolled, unabated and untreatable at present; no potential therapeutic drug is available yet for its treatment. The treatments of SARS-CoV-2 infected patients are based on the experiences gained from SARS-CoV and MERS-CoV infection during 2003. There is an urgent need for a suitable drug, i.e., readily available around the globe with low cost, highly efficacious with lesser side effects for the treatment of COVID-19. Corticosteroids are off-patent and are the part of WHO model list of essential medicines, smoothly accessible worldwide at a cheaper cost, in multiple formulations and various dosage forms. The WHO and several countries have approved dexamethasone as the first treatment shown to decline death rate in patients with COVID-19 requiring oxygen or ventilator assistance. The methylprednisolone, hydrocortisone and prednisolone are the other drugs belonging to the class of corticosteroids, that have shown significant results in the treatment of COVID-19 patients. The corticosteroids restrain the immune system or cytokine storm, which possibly imparts relief to patients, whose lungs are damaged by an intense immune response sometimes observed in critically ill COVID-19 patients. The corticosteroid can be used for all age of patients, i.e., children, adults, the elderly and pregnant or breastfeeding women. Current management strategies and guidelines published by NIH recommend the use of corticosteroids alone or in combination for moderate, severe and critical infected COVID-19 patients, who are hospitalized and require supplemental oxygen. However, the use of corticosteroids is strictly prohibited for asymptomatic/presymptomatic mild and moderate infections of COVID-19 patients, who require supplemental oxygen but who do not require oxygen delivery. Another opinion about use of corticosteroids in COVID-19 is somehow different, i.e., some scientific reports consider it controversial due to its enormous side effects such as increased mortality, slow clearance of viral RNA from the respiratory tract, obstructed clearance of viral RNA from blood, psychosis, induced diabetes, avascular necrosis in survivors, induced lung damage or shock. While considering the fact of their side effects, some scientific reports support the application of corticosteroids at low-to-average dose for a shorter duration as it reduces the death rate and minimizes the duration of hospitalization for seriously ill patients with COVID-19 without showing secondary infection and other adverse effects. In conclusion, corticosteroid drugs are beneficial in COVID-19 with some limitations; however, some additional clinical studies are required to establish their role in the treatment of COVID-19 patients.

## Future prospect and future research

WHO has approved dexamethasone as the first corticosteroid drug used for the treatment of critically ill patients with COVID-19 necessitating oxygen or ventilator assistance to reduce mortality. Methylprednisolone and hydrocortisone are other drugs belonging to the class of corticosteroids, which have exhibited promising results in lowering mortalities. The corticosteroids open the new door, and future research may concentrate on the development of known and novel corticosteroids analogs for the treatment of COVID-19 patients. The use of corticosteroid for the treatment of COVID-19 treatment is controversial, unclear needs more scientific evidence and may be studied further. The use of systemic corticosteroids for longer duration and its effect on death rate and functional consequence in COVID-19 patients is unknown and will be the subject of future investigations. The use of corticosteroids in combination with other potent drugs found effective against COVID-19 such as immunomodulators: interferon-β, peginterferon alpha-2a and -2b, tocilizumab and BMS-986253; antiviral drugs: favipiravir, remdesivir, ribavirin, triazavirin and umifenovir; antiparasitic drugs: chloroquine, hydroxychloroquine and mefloquine; antibiotics: teicoplanin, oritavancin, dalbavancin, monensin and azithromycin need to be studied to evaluate how these drugs interact/potentiate/decrease with effect of corticosteroids. Moreover, the effect and efficacy of corticosteroids also need to be established when used alone versus in combination with the aforementioned drugs. Additionally, scientists are also required to further study about various uncertainties including steroid preparation, dosing, maximum dose duration and corticosteroids effect on viral replication for finding out a better treatment option for COVID-19 patients.

## Data Availability

All the data generated and analyzed during the study are included in the manuscript.

## Abbreviations

WHO: World Health Organization
SARS-CoV-2: Severe Acute Respiratory Syndrome Coronavirus 2
MERS-CoV: Middle East Respiratory Syndrome Coronavirus
ICU: Intensive Care Unit
IV: Intravenous
COPD: Chronic Obstructive Pulmonary Disease
ARDS: Acute Respiratory Distress Syndrome
ECMO: Extracorporeal Membrane Oxygenation
CSS: Cytokine Storm Syndrome
CRP: C-Reactive Protein
IFN-γ: Interferon-Gamma
JAK: Janus Kinase
ONFH: Osteonecrosis of the Femoral Head
